# Increased exposure to acute thermal stress is associated with a non-linear increase in recombination frequency and an independent linear decrease in fitness in Drosophila

**DOI:** 10.1186/s12862-015-0452-8

**Published:** 2015-08-27

**Authors:** Savannah Jackson, Dahlia M. Nielsen, Nadia D. Singh

**Affiliations:** Department of Biological Sciences, North Carolina State University, Campus Box 7614, Raleigh, NC 27695 USA; Bioinformatics Research Center, North Carolina State University, Raleigh, USA

## Abstract

**Background:**

Meiotic recombination rate has long been known to be phenotypically plastic. How plastic recombination evolves and is maintained remains controversial; though a leading model for the evolution of plastic recombination rests on the tenet that organismal fitness and recombination frequency are negatively correlated. Motivated by the mounting evidence that meiotic recombination frequencies increase in response to stress, here we test for a negative correlation between fitness and recombination frequency. Specifically, the fitness-associated recombination model (FAR) predicts that if stress increases meiotic recombination frequency, then increasing exposure to stressful conditions will yield an increasing magnitude of the recombinational response, while concomitantly decreasing fitness.

**Results:**

We use heat shock as a stressor to test this prediction in *Drosophila melanogaster*. We find that increased exposure to heat shock conditions is associated with a non-linear increase in meiotic recombination frequency. We also find an independent effect of heat shock on organismal fitness, with fitness decreasing with increased duration of thermal stress.

**Conclusions:**

Our results thus support the foundation of the FAR model for the evolution of plastic recombination. Our data also suggest that modulating recombination frequency is one mechanism by which organisms can rapidly respond to environmental cues and confer increased adaptive potential to their offspring.

**Electronic supplementary material:**

The online version of this article (doi:10.1186/s12862-015-0452-8) contains supplementary material, which is available to authorized users.

## Background

Meiotic recombination serves two critical functions. First, genetic recombination is the primary mechanism by which proper disjunction of homologous chromosomes is ensured during meiosis. Thus, recombination is central to the preservation of genomic integrity between generations. Defects in meiotic recombination have catastrophic consequences for the fitness of progeny [1, 2], underscoring the vital function of homologous recombination for the maintenance of organismal fitness. From an evolutionary perspective, meiotic recombination has another important function, which is to create novel combinations of alleles at linked loci. In this way, recombination can both facilitate adaptation as well as enable populations to purge deleterious alleles [3, 4].

Creating adaptive potential and facilitating adaptation may be particularly important under fluctuating selective pressure. Consistent with this idea, it has been shown that recombination frequencies can increase in response to environmental changes. This plasticity in recombination frequency, or capacity of a single genotype to produce different rates of recombination in different environments, appears particularly strong in response to stress. For example, maternal age influences recombination rate in many species [5–15], and temperature affects recombination rate in Drosophila [5, 16–21]. Mating-associated stress also appears to increase recombination frequency in Drosophila [11], and social stress has been associated with increased recombination in rodents [22]. Nutrient stress is associated with increased recombination frequencies in both yeast [23] and Drosophila [24].

Although plastic recombination appears pervasive in nature, its evolution and maintenance remain unclear. Theoretical models have been developed to explain recombination rate plasticity, including the fitness-associated recombination (FAR) model [25]. Under this model, genetic modifiers of recombination rate are themselves plastic, and the rate of recombination caused by a modifier depends on the condition of the organism in which it is found. With this model, plastic recombination can persist in natural populations if organismal fitness and recombination are negatively correlated. Stress-associated recombination thus provides a plausible mechanism through which low fitness (stressed) individuals could produce a higher proportion of recombinant progeny, thereby yielding the requisite negative correlation between fitness and recombination rate necessary for the maintenance of plastic recombination under the FAR model. However, the FAR model appears to have limited tractability in diploids [26] and empirical tests of the FAR model are limited to a single study [27], which makes it difficult to evaluate the general plausibility of this model for natural populations.

Although it is increasingly clear that stress is associated with increased meiotic recombination frequency, it is yet unknown whether the duration of the stress itself is correlated with the magnitude of the increase in recombination frequency. If fitness and recombination rate negatively correlated as required by the FAR model, then one would predict that increasing exposure to stress—either via intensity or duration—would yield a greater increase in meiotic recombination frequency. However, this simple prediction of the FAR model has yet to be tested empirically. Here we build on the rich history of *Drosophila melanogaster* as a model for plastic recombination and specifically test whether increased exposure to stress yields an increasing and upwards perturbation in recombination frequency. We use acute heat shock as our model stress, which has reliably been shown to yield a significant increase in meiotic recombination frequency [20, 21, 27]. We also leverage our data to test the basic tenet of the FAR model, which is a purported negative correlation between organismal fitness and recombination frequency. Our results indicate that the increasing duration of exposure to heat-shock conditions yields an increasing magnitude of the recombinational response to treatment. Interestingly, although this response is monotonically increasing, it is quadratic rather than strictly linear in nature. Our data also indicate that heat shock treatment is associated with a decrease in organismal fitness, and that this decrease is independent of heat-shock-associated changes in recombination frequency. Our results thus support the foundation of the FAR model for the evolution of plastic recombination and further suggest that modulating recombination frequency is one mechanism by which organisms can rapidly respond to environmental cues and confer increased adaptive potential to their offspring, which may be particularly valuable in a variable environment.

## Methods

### Fly strains

The wild-type line used for this experiment, RAL_45, was randomly selected from the Drosophila Genetic Reference Panel [28]. This line has a standard chromosome arrangement. The double mutant *ebony rough* (*ero*) line was obtained from the Bloomington stock center (stock 496). These visible mutations are recessive and approximately 20.4 centimorgans (cM) apart on the genetic map [29].

### Experimental crosses

**Fig. 1:**
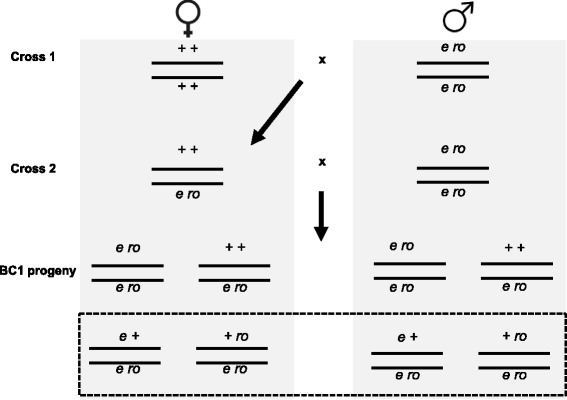
Schematic representation of the two-step crossing scheme using *ebony* (*e*) and *rough* (*ro*). Females used in each cross are shown on the left, males on the right. Boxed backcross 1 progeny (BC1) correspond to the two recombinant genotypes that can be visually identified using our screen

All crosses were conducted at 25° Celsius (C) on standard cornmeal/molasses Drosophila media (recipe available upon request) with a 12:12 light:dark cycle. A standard backcrossing scheme was used for this experiment (Fig. 1). In the first cross, virgin RAL_45 females and *e ro* males were mated in 8 ounce bottles. Twenty males and twenty females were used in each cross. This cross was conducted at 25 °C. Parental flies were cleared from these bottles after 5 days, and virgin doubly heterozygous females were collected within a 12-h window. After 24 h, these females were subject to control or heat-shock treatment. Females were thus 24-36 h at the time of treatment. For the heat-shock, we exposed flies to 35 °C for 6, 12, 18 and 24 h. After heat-shock, flies were returned to a temperature of 25 °C for the remainder of the 24 h treatment period. Thus, the 6-h treatment group was exposed to 35° for six hours, then returned to 25 °C for 18 h before being mated. An excess of virgin flies was heat shocked to account for fly death during experimental treatment, which was on the order of 15 %. Control flies were held at 25 °C for 24 h. Immediately following the 24-h treatment window, surviving treated and control females were then backcrossed to *ero* males in bottles; 40 females and 20 males were used in each replicate. Progeny were collected and scored for sex and the recombinant and non-recombinant phenotypes. This experiment was conducted in 2 batches, with 6 replicates per treatment in batch 1 and 9 replicates per treatment in batch 2.

### Statistical analysis

All statistical analyses were performed using the JMP® statistical software package (www.jmp.com). Recombination frequency was estimated separately for each replicate as [male(+*ro*) + male(*e*+) + female(+*ro*) + female(*e*+)]/(total males + total females). A Shapiro-Wilk test of normality fails to reject the null hypothesis that these recombination frequencies are normally distributed (*P* = 0.17), and we thus used parametric statistical approaches for data analysis. The effects of time and batch on both recombination frequency and number of offspring were tested separately by linear regression, fitting the model

$$ {Y}_{ijk}=\mu +{\beta}_1\mathrm{batc}{\mathrm{h}}_i+{\beta}_2\mathrm{t}\mathrm{i}\mathrm{m}{\mathrm{e}}_j+{\beta}_3\mathrm{t}\mathrm{i}\mathrm{m}{\mathrm{e}}_j^2+{\in}_{ijk} $$

where *Y* is either recombination frequency or total number of offspring. Batch (*i* = 1, 2) indicates whether the replicate came from the first or second iteration of this experiment, and time (*j* = 1, …, 5) denotes whether flies were subject to heat shock for 0, 6, 12, 18, or 24 h, and *k* indicates the replicate (for each batch and time).

## Results and discussion

### Robustness of recombination frequency estimation assay

A total of 31,229 flies were scored for this experiment. These flies were roughly evenly distributed among treatments, with between 16 % and 23 % of the total flies corresponding to each treatment. Within the control treatment, the average crossover frequency observed among replicates is 21.4 cM, which corresponds well to the published genetic map distance of 20.4 cM [29]. Given that our assay for estimating recombination frequency depends on visible markers, we first confirmed that, as previously suggested [29], there were no fitness effects associated with these mutations. To assess this, we test for a deviation from the expected 1:1 ratio of phenotype classes. Specifically, if there are no fitness effects associated with the *e* and *ro* mutations, we expect to see equal numbers of wild-type (++) and double-mutant progeny (*e ro*), and equal numbers of both recombinant phenotype classes (+*ro* and *e*+). We summed the progeny counts across all replicates within a given treatment and used a G-test for goodness of fit to test for viability defects associated with the marked chromosomes. The data are presented in Additional file 1: Table S1. For each treatment, we saw no significant deviation from the expected 1:1 ratio for either the ratio of two nonrecombinant phenotypes (++:*e ro*) or the ratio of the two recombinant phenotypes (*e*+:+*ro*) (Bonferroni-corrected *P* > 0.1, all tests). This suggests that any viability defects associated with the marked chromosome are too small in magnitude to markedly affect recombination frequency as estimated by our backcrossing assay. Coupled with the observation that crossover frequency observed in our control treatment aligns well with the expected map distance between these two markers, these data suggest that this recombination assay is both robust and accurate.

### Effects of heat-shock on recombination frequency

The observation that meiotic recombination frequency in Drosophila females increases in response to acute heat shock was first made fifty years ago [21]. Though this has been confirmed more recently [27], the timing of the recombinational response to heat shock appears quite variable. While it was initially shown that recombination frequency increases in response to heat shock, that increase did not manifest until 5 days after treatment [21]. In contrast, later work showed clearly that recombination frequency increases in the three-day window following treatment [27]. This difference is critical for understanding the mechanism underlying the observed increase in recombination frequency; while the 5 day delay between treatment and an increase in recombination is indicative of a meiotic origin of the increased crossing over [20, 21], an immediate effect of heat-shock points to an alternative mechanism such as transmission distortion as has been argued elsewhere [30]. This stems from the knowledge that *D. melanogaster* crossovers are initiated and resolved in developing oocytes 4-5 days before eggs are fertilized and laid [31, 32]. Thus, an increase in recombination frequency due to transmission distortion may manifest very soon after an environmental perturbation, while an increase in recombination frequency due to increased crossing-over during meiotic prophase is not expected to manifest for 4-5 days.

Another striking observation that emerges from the literature is the marked variation in the magnitude of the recombinational response to heat shock relative to controls, ranging from a 2-3 fold increase [21] to a 10-fold increase [27]. While some of this variation is likely due to the size of the interval used for estimating recombination frequency, which necessarily imposes limits on the maximum possible increase in recombination frequency one could observe, it may also be due in part to experimental differences in administering heat shock with respect to exposure temperature, exposure duration, and the age of the exposed females. To shed additional light on the timing of the recombinational response to heat shock and to test the hypothesis that the magnitude of the increase in recombination frequency in response to heat shock is dependent on the duration of treatment, we measured recombination frequency in females exposed to 35° for 0, 6, 12, 18, and 24 h. We only surveyed eggs laid in the first 5 days following exposure for recombination frequency estimation; any perturbation in recombination frequency observed in this period is likely not meiotic in origin but rather primarily due to transmission distortion.

**Fig. 2:**
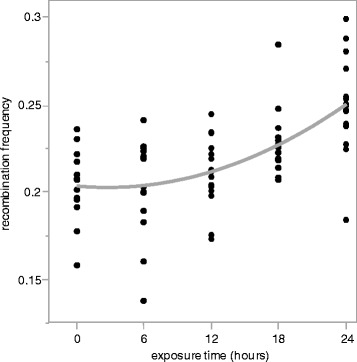
Recombination frequency increases quadratically across exposure time. Each point represents a replicate. The gray line is the regression fitting time (*P* = 3.6 × 10^−8^) and time*time (*P* = 0.021). For the purpose of illustration, recombination frequency estimates displayed here were adjusted for batch effects. Note that the y-axis does not start at 0

Results from our linear regression model indicate that both duration of exposure (‘time’) and ‘batch’ (see Materials and Methods) both significantly affect recombination frequency. The effect of batch (*P* = 0.0026 for recombination rate and 7.8 × 10^−4^ for number of offspring) is likely due to small variations in fly media and humidity conditions between the two batches. Our data confirm that recombination frequency increases in response to acute temperature stress. We further find that the magnitude of the increase in recombination frequency in heat-shocked females relative to control females significantly increases with increasing duration of heat-shock (Fig. 2). Interestingly, while there is a linear component to this relationship (*P* = 3.6 × 10^−8^), recombination frequency appears to increase quadratically across treatments and indeed, the time^2^ term in our linear model (see Materials and Methods) also significantly contributes to the variation in recombination frequency observed in this experiment (*P* = 0.021). Non-parametric analysis confirms a significant effect of exposure time on recombination frequency (*P* = 4.4 × 10^−6^, Kruskal-Wallis test).

Though previous work consistently indicated that heat shock is associated with an increase in recombination frequency, the extent to which such an effect could be observed in the first few days following treatment was inconsistent among studies [20, 21, 27]. That we observe a perturbation in recombination frequency so quickly after heat-shock has been administered strongly suggests that this perturbation is mediated at least in part by transmission distortion. A rapid change in recombination frequency in response to environmental conditions including heat shock, cold shock, and mating stress in Drosophila has been documented previously [11, 27], and this likely implicates transmission distortion as an underlying mechanism rather than an increase in crossing-over during meiosis as explained above. We hypothesize that the transmission distortion is mediated by an asymmetry in meiosis II, and future work will be aimed at testing this hypothesis.

Our data thus support the idea that Drosophila females can plastically alter their recombination fraction in rapid response to environmental conditions. The extent to which this is generalizable remains unknown. Future work will be aimed at determining the role of genetic background in the recombinational response to heat shock, as well as whether other acute or chronic stressors can perturb recombination frequency via transmission distortion. It will be particularly interesting from an evolutionary perspective to assess whether this transmission-distortion mediated increase in recombination frequency is observed in other taxa, as it would represent a powerful mechanism by which organisms could generate genetic diversity within their offspring in response to environmental conditions on an ecologically-relevant timescale.

Also unexpected was the non-linear relationship between the recombination frequency and the duration of exposure to heat shock conditions. That the relationship appears to be quadratic in nature may point to some feedback mechanism at the organismal level that serves to mediate the recombinational response to acute or chronic stress. However, it is important to note that in spite of the quadratic dependence of recombination frequency on exposure time, the increase in recombination frequency between the treatment with the longest exposure (24 h) and the control remains modest, at 25 %, in comparison to previous work which showed up to a 1000 % increase in recombination frequency [27]. Though this is certainly due in part to our choice of interval which, at 20 cM, only enables detection of increases in recombination frequency up to ~150 %, other genetic and environmental factors are likely to contribute to this as well. Previous work on the frequency of homologous recombination in somatic tissue of thermally-stressed plants illustrates clearly that the magnitude and direction of the change in homologous recombination frequency depends on temperature, the age at which the plants were exposed, and the duration of exposure [33]. As alluded to earlier, recombination frequency in Drosophila is remarkably plastic, varying in response to a number of factors including age, temperature, nutritional status, and genetic background [7, 9, 17, 19, 24, 27, 30, 34]. It therefore seems likely that some of the differences among studies are due to differences in environmental conditions.

Further work is required to assess the extent to which factors such as genetic background and maternal age mediate the magnitude of the recombinational response to stress. An interesting and open question is whether, for instance, phenotypic plasticity in recombination frequency varies as a function of maternal age. In addition, exploring the relationship between recombination frequency and the duration of exposures to other types of stressors will be of interest, and will illustrate the extent to which the quadratic dependence of recombination frequency on exposure time observed in the current study is generalizable. If this appears to be a canonical response, this may ultimately help in identifying signaling pathways underlying the organismal response to heat shock in particular or stress in general that lead to perturbations in recombination frequency.

### Effects of heat-shock on offspring production

The evolution of plastic recombination remains puzzling. As described earlier, one model for the evolution of plastic recombination is the fitness-associated recombination model, which was developed as a haploid model [25] and subsequently studied in the diploid case [26]. The foundation for this model is a negative correlation between organismal fitness and recombination frequency; such a correlation allows for all of the benefits of recombination (potentially bringing together favorable alleles to create higher-fitness haplotypes) without incurring the cost of recombination (destroying favorable combinations of alleles in high-fitness haplotypes). This correlation can manifest in several ways, including stress-associated recombination. Our results thus provide an opportunity to test whether the negative correlation between fitness and recombination frequency underlying the fitness-associated recombination model is supported in the case of acute thermal stress.

**Fig. 3:**
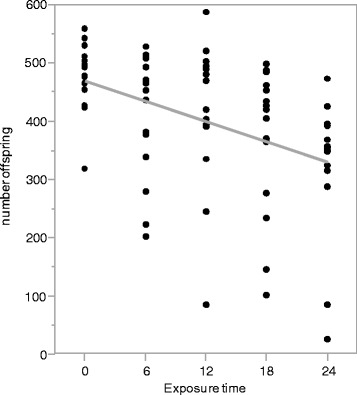
Number of offspring decreases across with increased exposure to heat shock conditions. The gray line is the regression fitting time (*P* = 2.4 × 10^−4^). For the purpose of illustration, values displayed here were adjusted for batch effects

**Fig. 4:**
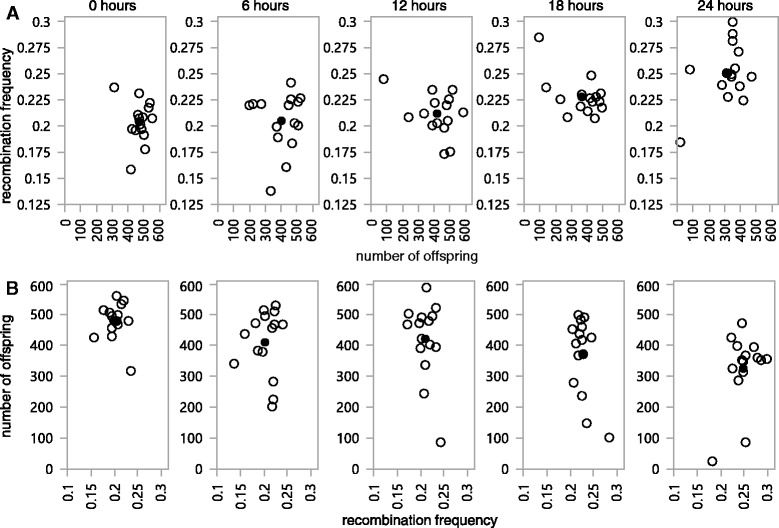
No correlation between recombination rate and number of offspring within time points. Open circles are individual replicates; filled circles are the mean values within time points. Random scatter is indicative of no correlation. In series A, the Y-axis is the recombination rate, where the increase with increased exposure can be seen by following the mean observations across time. In series B the axes are reversed so that number of offspring is along the Y-axis, and the decline with increased exposure time is apparent. Although recombination rate and number of offspring are negatively correlated overall, exposure time appears to be the effect driving changes in both factors independently, rather than recombination rate affecting number of offspring or vice versa

The general relationship between fitness and recombination in Drosophila appears far from straightforward. Early work found a strong, negative correlation between fitness and recombination frequency [35] based on natural, population-level genetic variation in both of these traits. More recent work exploiting a combination of wild-type and marked laboratory lines revealed no significant correlation between offspring production and recombination frequency [10]. Recombination and fitness have also been found to be negatively correlated in heat-shocked flies, but not in control, mating-stressed, or cold-stressed flies [27]. Our results show that fitness (as measured by progeny production) significantly decreases with increasing duration of exposure to heat shock conditions (*P* = 2.1 × 10^−4^; Fig. 3). Non-parametric analysis confirms this finding (*P* = 0.0007, Kruskal-Wallis test). The average progeny count for control flies was 487 progeny/bottle, with average progeny counts of 450, 418, 386, and 341 for the 6, 12, 18, and 24 h heat shock treatment flies, respectively (Fig. 4). This corresponds to a ~30 % decrease in progeny production between the control and 24-h treated flies. The increase in recombination frequency with increased exposure (Fig. 2) coupled with the decrease in progeny production with increased exposure (Fig. 3) yields the requisite negative correlation between fitness and recombination frequency required in the fitness-associated recombination model. However, it is important to note that while fitness and recombination are negatively correlated, this relationship does not appear causal. Rather, exposure time appears to be independently driving both the increase in recombination frequency and the decrease in offspring production. This is evidenced by the observation that within any given treatment, there is no significant correlation between recombination frequency and offspring production (Fig. 4). If recombination frequency was functionally linked to offspring production or vice versa, then we would expect that within an exposure treatment, changes in recombination frequency would be associated with changes in fitness, which is not what is observed. Thus, the overall correlation observed between fitness and recombination appears driven by the third variable, exposure time.

Although our findings support the correlation between fitness and recombination frequency that is central to the fitness-associated-recombination model [25], it is important to note that this does not necessarily suggest that this model is likely to be applicable for natural populations of Drosophila. Theoretical work illustrates that the fitness-associated recombination is less likely to evolve in diploids relative to the haploid case. Specifically, the evolution of fitness-associated recombination in diploids requires 1) a mechanism by which a recombination modifier is provided information about the haplotype on which it resides, 2) cis-trans epistasis, or 3) maternal effects on fitness [26]. While there is certainly evidence in support of maternal effects on fitness in Drosophila (e.g. [36]), it is yet unknown whether stress-associated reductions in fitness such as those observed in the current study also yield maternal fitness effects.

## Conclusions

We leveraged classical genetic approaches to experimentally test a prediction of the fitness-associated recombination model for the evolution of plastic recombination. We used heat shock as a model stressor, and *Drosophila melanogaster* as a model system. Our data strongly support the hypothesis that increasing exposure to stress yields a greater increase in meiotic recombination frequency. This increase in recombination in response to heat shock appears driven by transmission distortion given how quickly it manifests relative to the environmental perturbation. We also find that the relationship between heat shock exposure and recombination frequency is not strictly linear and rather, appears quadratic in nature. The biological significance of the shape of this relationship remains unknown. Our data also support an effect of heat shock on progeny production; this effect is independent from the effect of heat shock on recombination frequency. Our data thus provide empirical support for the fitness-associated recombination model, though continued work is needed in the future to understand the applicability of this model to the evolution of plastic recombination in natural populations.

## Additional file

Additional file 1: Table S1. Progeny counts for each phenotype class in each treatment group. (DOC 30 kb)

## Abbreviations

FAR: Fitness-associated recombination
cM: Centimorgan
