# Supplementary material for: Increased exposure to acute thermal stress is associated with a non-linear increase in recombination frequency and an independent linear decrease in fitness in Drosophila
Source: BMC Evol Biol. 2015 Aug 27;15:175. doi: 10.1186/s12862-015-0452-8 (PMC4551699; doi:10.1186/s12862-015-0452-8)
Supplement: Additional file 1: Table S1. — Progeny counts for each phenotype class in each treatment group. (DOC 30 kb) [file 12862_2015_452_MOESM1_ESM.doc]

Supplementary Table 1: Progeny counts for each phenotype class in each treatment group.

| Duration of heat shock (hrs) | ++ | *e ro* | + *ro* | e+ |
| --- | --- | --- | --- | --- |
| 0 | 2995 | 2760 | 784 | 765 |
| 6 | 2766 | 2514 | 770 | 696 |
| 12 | 2544 | 2341 | 655 | 729 |
| 18 | 2341 | 2108 | 652 | 689 |
| 24 | 1937 | 1846 | 670 | 667 |
